# microRNA-9 and -29a regulate the progression of diabetic peripheral neuropathy *via* ISL1-mediated sonic hedgehog signaling pathway

**DOI:** 10.18632/aging.103230

**Published:** 2020-06-16

**Authors:** Qin Sun, Jun Zeng, Yang Liu, JingYan Chen, Qing-Cui Zeng, Yan-Qiu Chen, Li-Li Tu, Ping Chen, Fan Yang, Min Zhang

**Affiliations:** 1 Department of Geriatrics, Sichuan Academy of Medical Sciences and Sichuan Provincial People’s Hospital, Chengdu 610072, P. R. China; 2 Chengdu Medical College, Chengdu 610500, P. R. China; 3 Department of Neurology, People’s Hospital of Chongqing Yubei, Chongqing 401120, P. R. China; 4 Department of General Medicine, Sichuan Academy of Medical Sciences and Sichuan Provincial People's Hospital, Chengdu 610072, P. R. China

**Keywords:** microRNA-9, microRNA-29a, diabetic peripheral neuropathy, Insulin gene enhancer binding protein-1, sonic hedgehog signaling pathway

## Abstract

In this study, we tested the hypothesis that overexpression of miR-9 and miR-29a may contribute to DPN development and progression. We performed a meta-analysis of miR expression profile studies in human diabetes mellitus (DM) and the data suggested that miR-9 and miR-29a were highly expressed in patients with DM, which was further verified in serum samples collected from 30 patients diagnosed as DM. Besides, ISL1 was confirmed to be a target gene of miR-9 and miR-29a. Lentivirus-mediated forced expression of insulin gene enhancer binding protein-1 (ISL1) activated the sonic hedgehog (SHH) signaling pathway, increased motor nerve conduction velocity and threshold of nociception, and modulated expression of neurotrophic factors in sciatic nerves in rats with DM developed by intraperitoneal injection of 0.45% streptozotocin, suggesting that ISL1 could delay DM progression and promote neural regeneration and repair after sciatic nerve damage. However, lentivirus-mediated forced expression of miR-9 or miR-29a exacerbated DM and antagonized the beneficial effect of ISL1 on DPN. Collectively, this study revealed potential roles of miR-9 and miR-29a as contributors to DPN development through the SHH signaling pathway by binding to ISL1. Additionally, the results provided an experimental basis for the targeted intervention treatment of miR-9 and miR-29a.

## INTRODUCTION

Diabetes mellitus (DM) is one of the most serious chronic diseases across the world, affecting millions of lives [1]. DM is associated with a wide spectrum of neuropathy syndromes, ranging from a mild asymptomatic distal sensory neuropathy to a severe and disabling radiculoplexus neuropathy [2]. Indeed, neuropathy is one of the most common chronic complications of DM. Diabetic neuropathy is a heterogeneous disease characterized by a complicated pathophysiology, with great impact on the peripheral nervous system [3]. It has been previously demonstrated that the pathogenesis of diabetic peripheral neuropathy (DPN) is affected by oxidative stress, neuroinflammation and apoptosis [4, 5]. Clinical manifestations of DPN include increased vibration and thermal perception thresholds that progress to sensory loss, which occur in addition to degeneration of all fiber types in the peripheral nerve fibers [6]. Painful diabetic neuropathy, a known syndrome of DPN, greatly affects the quality of life of patients with DM and brings great financial burden, while currently available treatments intended fail to prevent the development of DPN [7]. In addition, DPN can lead to foot ulcers, accounting for the increasing number of amputations in patients with DM [8]. Therefore, to improve the life quality of patients with DM, it is essential to find an effective prevention and treatment for DPN.

microRNAs (miRs) are small non-coding RNAs that play a significant role in various biological processes, including complex metabolic processes (energy and lipid metabolism) which are associated with DM [9]. miR-9 plays a critical role in the regulation of neurodevelopment, and is overexpressed in the vertebrate nervous system in rat models [10]. Notably, miR-9 is closely associated with insulin secretion and beta cell development and function [11]. miR-29a, a member of the miR-29 family, is implicated in the pathogenic mechanism of diabetic nephropathy and insulin resistance, and has the potential to serve as an alternative biomarker for diabetic nephropathy in patients with type 2 diabetes mellitus (T2DM) [12]. In addition, miR-29a is involved in various physiological and pathological processes, including DM, osteoblastic differentiation, cardiac fibrosis and tumorigenesis [13]. According to the target prediction program available on miRanda and TargetScan (http://www.targetscan.org/vert_71/), insulin gene enhancer binding protein-1 (ISL1) was a putative direct target gene of miR-9 and miR-29a. ISL1 is an important transcription factor, which belongs to the LIM homeobox gene family, mainly identified in adult islet endocrine cells and the central nervous system [14]. Furthermore, ISL1 is thought to have particular involvement in the formation of the exocrine and endocrine compartments of the pancreas, as well as in mediation of insulin secretion and metabolism in T2DM [15, 16]. Sonic hedgehog (SHH) is the most abundantly expressed among the three vertebrate hedgehog homologs, and plays important roles in the central nervous system, cell differentiation, and embryonic development [17]. A previous study has found that SHH contributes to cavernous nerve regeneration and repair following crush injuries [18, 19]. Moreover, various types of nerve injuries are known to be closely related to DPN [20]. SHH protein has the ability to reconstruct nerve function and cause arteriogenesis in diabetic neuropathy [21]. Besides, the SHH signaling pathway may play a role in peripheral nerve regeneration through modulation of myelin deterioration [22]. However, the roles of miR-9 and -29a involving ISL1 and the SHH signaling pathway are still to be investigated in DPN. Therefore, this study aims to elucidate the effect of miR-9/miR-29a on DPN through the SHH signaling pathway via ISL1.

## RESULTS

### miR-9 and -29a are highly expressed in sciatic nerves of rats with DM and in peripheral blood of patients with DM

**Figure 1:**
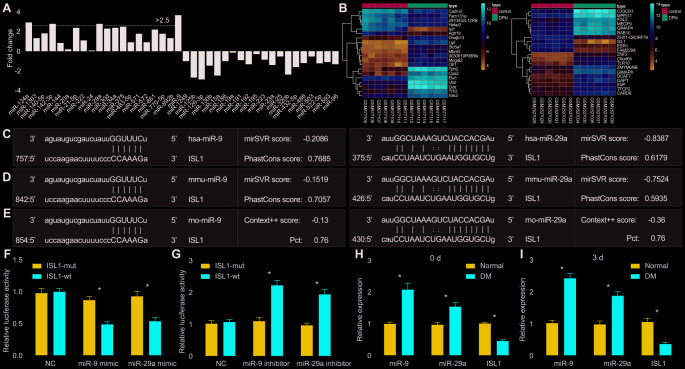
**miR-9 and miR-29a are identified with high expression in DM, and both can bind to 3’UTR of ISL1.** (**A**) The fold-change values of miRs calculated according to 16 included studies. Log_2_ Fold change of miR = Log_2_ (miR expression in DM) – Log_2_ (miR expression in NC). Fold change > 2 is indicative of upregulated miR in DM. Fold change < 0 is indicative of downregulated miR in DM. (**B**) The expression of ISL1 in microarray datasets GSE27382 (mouse sciatic nerves) and GSE95849 (human peripheral blood). (**C**) ISL1 was a putative target gene of both miR-9 and miR-29a in humans according to the target prediction program available on miRanda. (**D**) ISL1 was a putative target gene of both miR-9 and miR-29a in mice according to target prediction program available on miRanda. (**E**) ISL1 was a putative target gene of both miR-9 and miR-29a in rats according to target prediction program available on TargetScan. (**F**, **G**) The luciferase activity determination in HEK293T cells indicated that ISL1 was indeed a target gene of both miR-9 and miR-29a. (**H**) The expression of ISL1, miR-9 and miR-29a in sciatic nerves after streptozotocin induction on the 0 day determined by RT-qPCR. (**I**) The expression of ISL1, miR-9 and miR-29a in sciatic nerves after streptozotocin induction on the 3^rd^ day determined by RT-qPCR (n = 8). Luciferase activity and gene expression were measurement data and expressed as mean ± standard deviation. Luciferase activity was analyzed by two-way analysis of variance; gene expression was analyzed by *t* test; the experiment was repeated three times independently; * *p* < 0.05 *vs.* the normal or NC group.

Data extraction from 16 literatures [9, 23–37] was carried out in order to calculate the fold change value of each miR. Additionally, the expression patterns of miRs in peripheral blood of patients with DM and healthy participants were compared. It was found that miR-9, miR-124a, miR-142-3p and miR-29a were highly expressed in peripheral blood of patients with DM and poorly expressed in peripheral blood of healthy participants (*p* < 0.05) (Figure 1A). Subsequently, among the poorly-expressed genes in DM as revealed from microarray datasets GSE27382 and GSE95849, ISL1 expression presented significant changes, and thus ISL1 was selected for further analysis (Figure 1B).

The miRanda website predicted that ISL1 was the target gene of miR-9 and miR-29a in human subjects (Figure 1C). Meanwhile, according to prediction results available on miRanda and TargetScan websites, ISL1 was revealed as a putative target gene of miR-9 and miR-29a both in mice and rats (Figure 1D, 1E). A dual luciferase reporter gene assay further validated that ISL1 was indeed the target gene of miR-9 and miR-29a. Compared with the negative control (NC) group, the miR-9 mimic and miR-29a mimic groups with transfection of ISL1-wild type (wt) plasmid presented with significantly decreased luciferase activity (*p* < 0.05), while there were no obvious changes in the luciferase activity of the both groups after transfection of ISL1-mutant (mut) (*p* > 0.05) (Figure 1F). Moreover, miR-9 inhibitor and miR-29a inhibitor resulted in significantly enhanced luciferase activity of ISL1-wt (*p* < 0.05), while there was no significant difference regarding the luciferase activity of ISL1-mut (*p* > 0.05) (Figure 1G). These results indicated that miR-29a and miR-9 specifically bound to ISL1.

After the establishment of a DM rat model, miR-9 and miR-29a were upregulated and ISL1 was downregulated in sciatic nerves of DM rats compared with those of normal rats (*p* < 0.01) (Figure 1H). To further explore whether acute hyperglycemia was responsible for the expression alternation of miR-9, miR-29a and ISL1, reverse transcription quantitative polymerase chain reaction (RT-qPCR) was performed to determine the expression of miR-9, miR-29a and ISL1 immediately after DM model development using injection of streptozotocin. Results showed that expression of miR-9 and miR-29a was significantly higher and ISL1 expression was significantly lower in sciatic nerves of DM rats in comparison to those of normal rats (*p* < 0.01). However, no significant difference was witnessed between the 0 day and the 3^rd^ day after model development (Figure 1I), suggesting that acute hyperglycemia was not accountable for the expression changes of related genes. Therefore, these findings indicated that miR-9 and miR-29a were expressed highly in sciatic nerves of DM rats and in peripheral blood of patients with DM, and that ISL1 was the target gene of miR-9 and miR-29a.

### miR-9 and -29a inhibit the activation of SHH signaling pathway via ISL1

**Figure 2:**
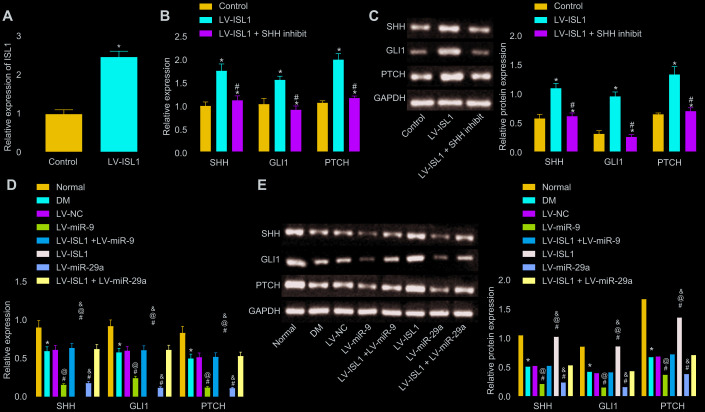
**miR-9 and miR-29a regulate ISL1-mediated SHH signaling pathway.** (**A**) The relative expression of ISL1 after lentiviral infection determined by RT-qPCR. (**B**) mRNA expression of SHH, GLI1, and PTCH in serum of rats after lentiviral infection detected by RT-qPCR. (**C**) The protein bands and expression of SHH, GLI1, and PTCH in serum of rats after lentiviral infection normalized to GAPDH detected by Western blot analysis; * *p* < 0.05 *vs.* the LV-NC group; # *p* < 0.05 *vs.* the LV-ISL1 group. (**D**) The effect of miR-9 and miR-29a on mRNA expression of SHH, GLI1, and PTCH in serum of rats detected by RT-qPCR. (**E**) The effect of miR-9 and miR-29a on protein bands and expression of SHH, GLI1, and PTCH in serum of rats normalized to GAPDH detected by Western blot analysis; * *p* < 0.05 *vs.* the normal group; # *p* < 0.05 *vs.* the DM group; @ *p* < 0.05 *vs.* the LV-ISL1 + LV-miR-9 group; & *p* < 0.05 *vs.* the LV-ISL1 + LV-miR-29a group. Data were measurement data and expressed as mean ± standard deviation; *t* test was performed for pairwise comparison; one-way analysis of variance was performed for multiple-group comparison; n = 8; the experiment was repeated three times independently.

Following lentiviral infection, RT-qPCR was carried out to detect the infection efficiency in sciatic nerves of rats. Results revealed significantly higher ISL1 expression in the lentiviral vector (LV)-ISL1 group compared with the LV-NC group (*p* < 0.05) (Figure 2A). Then, RT-qPCR and Western blot analysis were conducted in order to detect the mRNA and protein expression of the SHH signaling pathway and neurotrophic factor-related genes in the rat serum, respectively. After lentiviral infection, the results showed that the LV-ISL1 group exhibited obviously higher expression of SHH, glioma-associated oncogene homolog 1 (GLI1) and patched tumor suppressor gene (PTCH) than the LV-NC group. Compared with the LV-ISL1 group, the LV-ISL1 + SHH inhibit group exhibited significantly decreased expression of SHH, GLI1 and PTCH (*p* < 0.05), and did not differ obviously when compared with the LV-NC group (*p* > 0.05) (Figure 2B, 2C). These findings suggested that inhibition of the SHH signaling pathway and overexpression of ISL1 did not affect the downstream of the SHH signaling pathway, and ISL1 positively mediated the SHH signaling pathway. The expression of SHH, GLI1 and PTCH was reduced in the DM group, relative to the normal group. When compared with the LV-NC and LV-ISL1 + LV-miR-9 groups, the LV-ISL1 group exhibited notably increased expression of SHH, GLI1 and PTCH, while the LV-miR-9 group exhibited evidently reduced expression of these markers (*p* < 0.05). It was suggested that ISL1 exerted an antagonistic effect on miR-9, and that miR-9 negatively regulated ISL1 to affect the SHH signaling pathway (Figure 2D, 2E). Corresponding results for miR-29a were the same as those found for miR-9. Thus, both miR-29a and miR-9 targeted ISL1, and further regulated the downstream pathway of ISL1.

### miR-9 and -29a decrease nociception threshold and peripheral nerve conduction velocity

**Figure 3:**
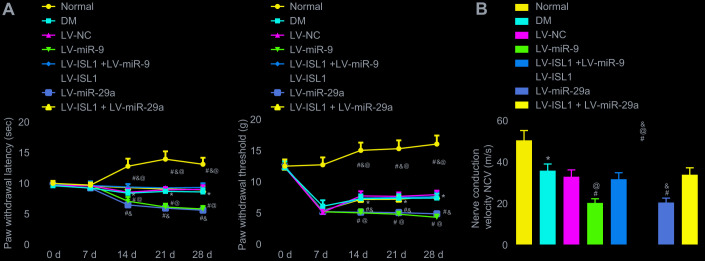
**miR-9 and miR-29a reduce peripheral nerve conduction velocity and nociception threshold in rats with DM.** (**A**) The paw withdrawal latency and paw withdrawal threshold of normal rats and rats with DM following lentiviral infection. (**B**) The nerve conduction velocity of normal rats and rats with DM following lentiviral infection on the 28^th^ day after model development; * *p* < 0.05 *vs.* the normal group; # *p* < 0.05 *vs.* the DM group; @ *p* < 0.05 *vs.* the LV-ISL1 + LV-miR-9 group; & *p* < 0.05 *vs.* the LV-ISL1 + LV-miR-29a group. Results were measurement data and expressed as mean ± standard deviation; the repeated measures analysis of variance was used to analyze data at different time points; n = 8; the experiment was repeated three times independently.

The nerve conduction velocity was measured after 4 weeks using the aforementioned methods, and the nociception threshold, represented by paw withdrawal latency and paw withdrawal threshold, was determined weekly in each group. After establishing the DM rat model, the rats showed significantly increased feed intake and urine volume, in addition to decreased exercise, spontaneous activity and reactions. After infection with lentivirus for rats, withdrawal thermal latency (WTL) and withdrawal mechanical threshold (WMT) were measured and recorded on a weekly basis. As shown in Figure 3A, rats in the DM group showed obviously decreased nociception threshold (*p* < 0.05). In comparison with the LV-NC and LV-ISL1 + LV-miR-9 groups, the LV-miR-9 group exhibited a notably decreased nociception threshold, while the LV-ISL1 group presented with increased nociception threshold (*p* < 0.05). miR-29a and miR-9 exhibited the same regulatory effect on the ISL1 gene. On the 28^th^ day after model development, the nerve conduction velocity was measured in each group. As shown in Figure 3B, the LV-ISL1 group exhibited significantly improved nerve conduction velocity, while the LV-miR-9 and LV-miR-29a groups showed reduced nerve conduction velocity. The LV-ISL1 + LV-miR-9 and LV-ISL1 + LV-miR-29a groups exhibited higher nerve conduction velocity relative to the LV-miR-9 and LV-miR-29a groups, however, the velocity was slower relative to the LV-ISL1 group (*p* < 0.05). These findings suggested that the miR-9 and miR-29a decreased nerve conduction velocity and nociception threshold.

### miR-9 and -29a aggravate disease activity in rats with DM by decreasing ISL1

**Figure 4:**
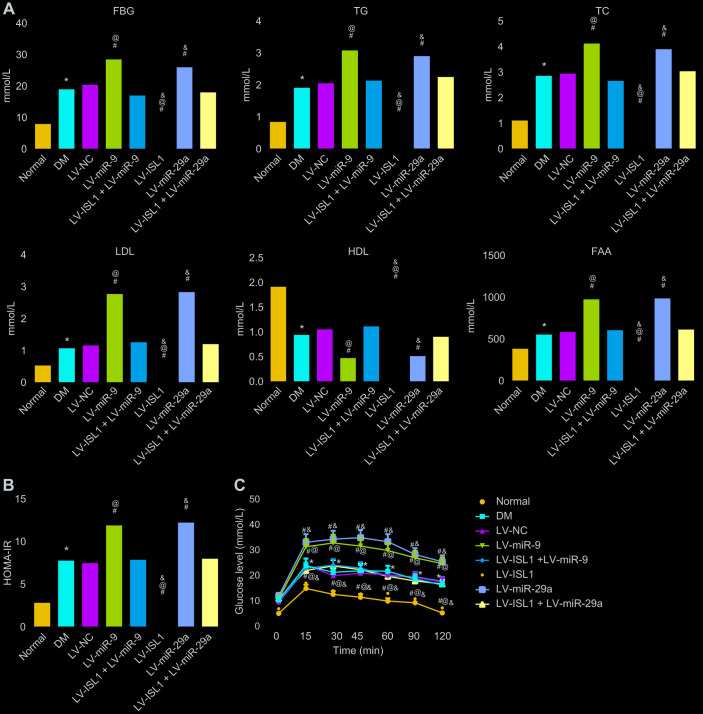
**miR-9 and miR-29a affect disease manifestation of DM in rats.** (**A**) The expression of FBG, TG, TC, LDL, FFA and HDL in the serum of normal rats and rats with DM following lentiviral infection. (**B**) HOMA-IR level of normal rats and rats with DM following lentiviral infection. (**C**) The glucose level of normal rats and rats with DM following lentiviral infection detected by OGTT assay; * *p* < 0.05 *vs.* the normal group; # *p* < 0.05 *vs.* the DM group; @ *p* < 0.05 *vs.* the LV-ISL1 + LV-miR-9 group; & *p* < 0.05 *vs.* the LV-ISL1 + LV-miR-29a group. Results were measurement data and expressed as mean ± standard deviation; one way analysis of variance was used to analyze data among multiple groups; n = 8; the experiment was repeated three times independently.

At 4 weeks after lentivirus infection in rats, rat serum was obtained in order to test blood glucose and lipid content. The result showed that rats with DM exhibited higher levels of fasting blood glucose (FBG), triglycerides (TG), total cholesterol (TC), low density lipoprotein (LDL), free fatty acids (FFA), and homeostasis model assessment of insulin resistance (HOMA-IR), yet lower high density lipoprotein (HDL) level than normal rats (*p* < 0.05). In comparison with the LV-NC and LV-ISL1 + LV-miR-9 groups, the LV-miR-9 group exhibited markedly increased levels of FBG, TG, TC, LDL, FFA and HOMA-IR, and notably decreased HDL; while the LV-ISL1 group exhibited significantly decreased levels of FBG, TG, TC, LDL, FFA and HOMA-IR, and increased HDL (*p* < 0.05). Thus, rats in the LV-ISL1 group showed improved biochemical indexes while the rats in the LV-miR-9 group exhibited aggravated DM (Figure 4A, 4B). miR-29a presented with the same results as miR-9.

The results of oral glucose tolerance test (OGTT) (Figure 4C) showed that at the 12^th^ week, blood glucose level of rats with DM was obviously higher than in normal rats (*p* < 0.05). At 15, 30, 45, 60, 90 and 120 min respectively, compared with the LV-NC and LV-ISL1 +LV-miR-9 groups, the blood glucose level was elevated in the LV-miR-9 group, while decreased in the LV-ISL1 group (*p* < 0.05). Transfection with miR-29a gave similar results as miR-9. Thus, downregulating miR-9 and miR-29a increased blood glucose and blood lipid-related indexes and inhibited the development of DM.

### miR-9 and -29a aggravate structure lesions and increase aldose reductase (AR) activity of sciatic nerves in rats with DM via decreasing ISL1

**Figure 5:**
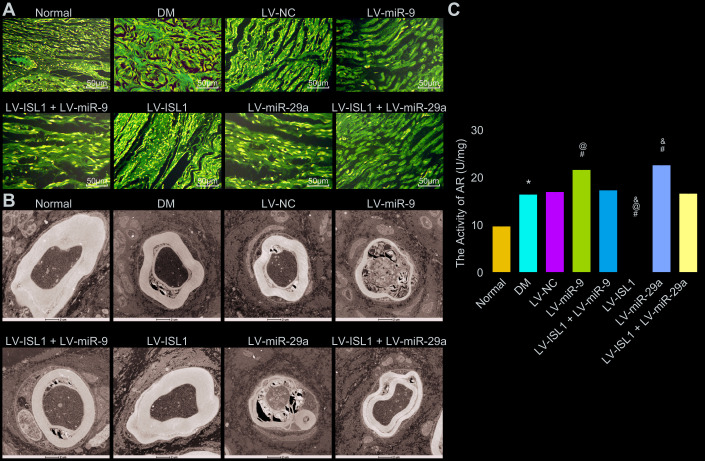
**miR-9 and miR-29a aggravate the structural pathological changes of the sciatic nerves in rats with DM.** (**A**) The sciatic nerves of normal rats and rats with DM following lentiviral infection detected by HE staining (× 200). (**B**) The sciatic nerve detection of normal rats and rats with DM following lentiviral infection examined by electron microscope (× 10000). (**C**) AR activity of sciatic nerve detection in normal rats and rats with DM following lentiviral infection; * *p* < 0.05 *vs.* the normal group; # *p* < 0.05 *vs.* the DM group; @ *p* < 0.05 *vs.* the LV-ISL1 + LV-miR-9 group; & *p* < 0.05 *vs.* the LV-ISL1 + LV-miR-29a group. Results were measurement data and expressed as mean ± standard deviation; one way analysis of variance was used to analyze data among multiple groups; n = 8; the experiment was repeated three times independently.

The pathological morphology of sciatic nerves was observed using electron microscopy with hematoxylin-eosin (HE) staining. The results (Figure 5A) showed that the normal rats displayed continuous and closely arranged sciatic nerve fibers and uniformly distributed fiber density, while in the DM and LV-NC group, the sciatic nerve fibers were prone to denaturation and fractures, with a relatively loose arrangement and large gaps, the density of myelinated nerve fibers was uneven and significantly, in addition to unevenly-stained and thin myelin sheaths. Compared with the LV-NC group, the LV-ISL1 group presented with compact sciatic nerve fibers with uniform distribution and neat arrangement, in addition to half-moon-shaped Schwann cells with complete structure, even thickness, and coloring of myelin sheaths. The LV-miR-9 and LV-miR-29a groups exhibited significantly increased denaturation and fractures of the sciatic nerve fibers, with loose structure, thinner myelin sheaths and more uneven coloring. There were no significant differences between the LV-ISL1 + LV-miR-9 group and the LV-ISL1 + LV-miR-29a group, wherein much denaturation and fractures of the sciatic nerve fibers were observed in addition to loose structure, thinner myelin sheaths, and uneven coloring. Electron microscope observation (Figure 5B) revealed Schwann cells with normal and complete cell structure, large and round karyon, as well as a well-structured medullary pin of the transverse section of the sciatic nerves in the normal rats. However, the DM and LV-NC groups presented with degeneration and necrosis of Schwann cells, cell debris, disordered lamellar structure and absent mitochondria. In comparison with the LV-NC group, the LV-ISL1 group had a well-structured, dense and uniform medullary pin of the transverse section of the sciatic nerves, appearing as a concentric circular lamellar structure with bright and dark patches; Schwann cells presented a normal and complete structure, with large round nuclei, and an abundance of euchromatin; neurofilaments, microfilaments, and microtubules, with axonal mitochondria having a regular arrangement and complete structure. However, in the LV-miR-9 and LV-miR-29a groups, the medullary pin of the transverse section of the sciatic nerves showed significantly separated lamellar structures, disordered arrangement between layers, a large number of vacuoles and fissures, axonal atrophy and thinning, absent mitochondria, along with increased denaturation and fractures of the sciatic nerve fibers, which had loose structure, thinner myelin sheaths, and uneven coloring. There were no significant differences between the LV-NC group and the LV-ISL1 + LV-miR-9/LV-miR-29a group. By means of pathological observations, miR-9 and miR-29a overexpression damaged the sciatic nerve structure, and ISL1 improved the sciatic nerve injury in rats, indicating an antagonistic relationship between ISL1 and miR-9/miR-29a, and that manipulation of miR-9 and miR-29a expression *in vivo* could alter the sciatic nerve status in rats.

Next, AR activity of rat sciatic nerves was further measured and the results (Figure 5C) showed that DM rats exhibited higher AR activity of sciatic nerves than in normal rats (*p* < 0.05). Compared with the LV-NC and LV-ISL1 + LV-miR-9 groups, AR activity of sciatic nerves was increased in the LV-miR-9 group yet decreased in the LV-ISL1 group (*p* < 0.05). The detection result of miR-29a was the same as miR-9. Hence, silenced miR-9 and miR-29a contributed to reduced AR activity of sciatic nerves in DM model rats.

### miR-9 and -29a affect neural regeneration after repair of sciatic nerve defect in rats with DM

**Figure 6:**
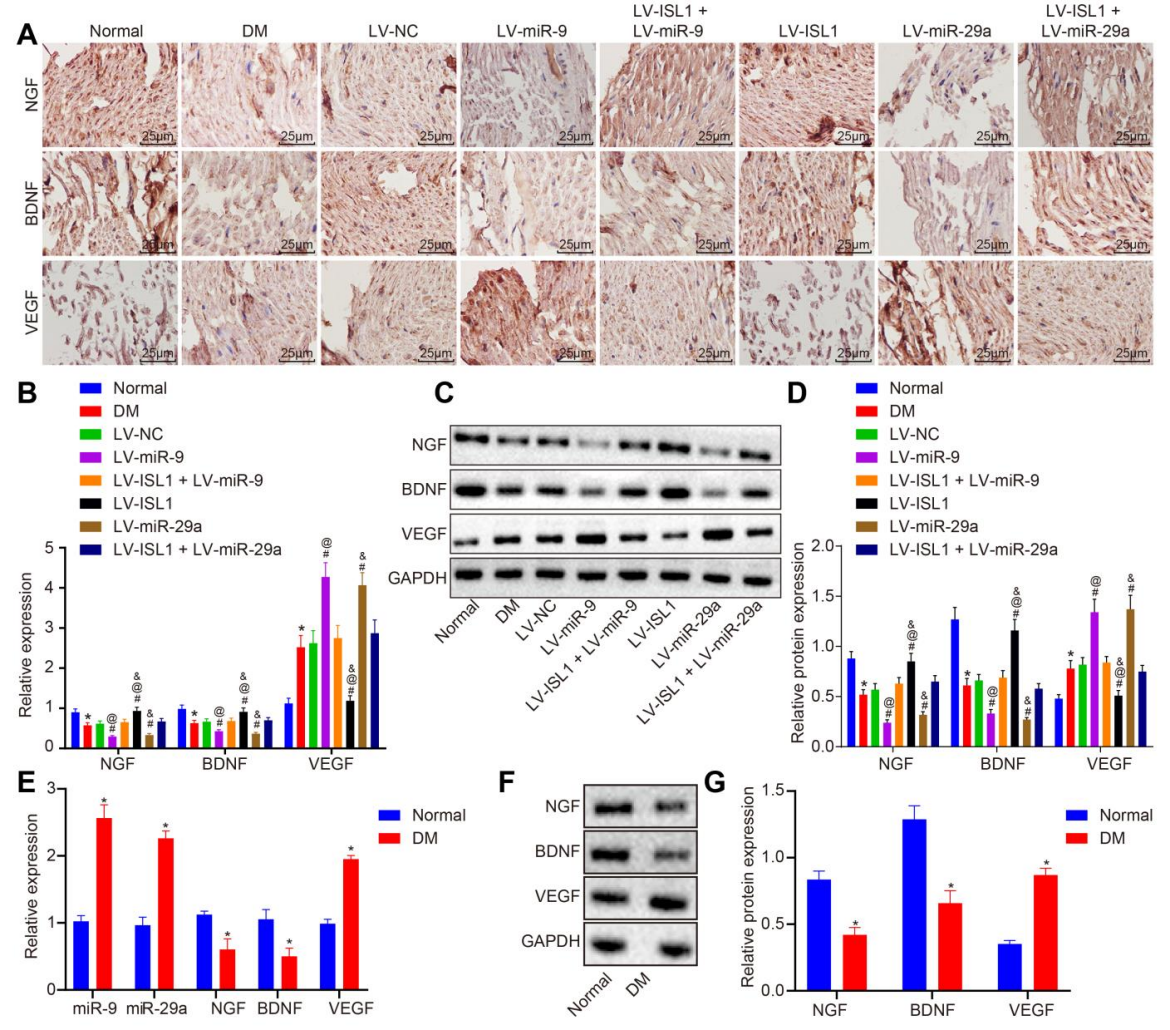
**miR-9 and miR-29a affect neural regeneration after repair of sciatic nerve defect in rats and patients with DM.** (**A**) The positive expression of NGF, BDNF and VEGF in sciatic nerve tissues of normal rats and rats with DM following lentiviral infection detected by immunohistochemistry (× 400) (n = 8). (**B**) mRNA expression of NGF, BDNF and VEGF in sciatic nerve tissues of normal rats and rats with DM following lentiviral infection detected by RT-qPCR (n = 8). (**C**, **D**) The protein expression of NGF, BDNF and VEGF in sciatic nerve tissues of normal rats and rats with DM following lentiviral infection normalized to GAPDH measured by Western blot analysis, with protein band assessed (n = 8). (**E**) mRNA expression of NGF, BDNF and VEGF in patients with DM detected by RT-qPCR (n = 30). (**F**, **G**) The protein expression of NGF, BDNF and VEGF in patients with DM normalized to GAPDH measured by Western blot analysis, with protein band assessed (n = 30); * *p* < 0.05 *vs.* the normal group; # *p* < 0.05 *vs.* the DM group; @ *p* < 0.05 *vs.* the LV-ISL1 + LV-miR-9 group; & *p* < 0.05 *vs.* the LV-ISL1 + LV-miR-29a group. Results were measurement data and expressed as mean ± standard deviation; one way analysis of variance was used to analyze data among multiple groups; n = 8; the experiment was repeated three times independently.

Nerve growth factor (NGF), brain-derived neurotrophic factor (BDNF), and vascular endothelial growth factor (VEGF) are neurotrophic factors, which promote the survival, growth, and differentiation of nerves. Immunochemistry was employed in order to detect the expression of NGF, BDNF and VEGF in sciatic nerve tissues of rats in each group. As shown in Figure 6A, NGF, BDNF and VEGF were mainly distributed in the cytoplasm of the cells. The LV-ISL1 group exhibited relatively increased expression of NGF and BDNF in the sciatic nerves, which showed strong positive expression, but obviously decreased positive expression rate of VEGF. Compared with the normal rats, the DM rats exhibited decreased positive expression of NGF and BDNF, yet increased expression of VEGF. The LV-miR-9 and the LV-miR-29a groups presented with evidently decreased positive expression of NGF and BDNF with increased positive expression of VEGF in the sciatic nerves. The LV-NC, LV-ISL1 + LV-miR-9 and LV-ISL1 + LV-miR-29a groups exhibited higher positive expression of NGF and BDNF as well as lower positive expression of VEGF compared with the LV-ISL1 group, while lower positive expression of NGF and BDNF as well as higher positive expression rate of VEGF relative to the LV-miR-9 and the LV-miR-29a groups.

To further verify the expression of neurotrophic factors in the sciatic nerves, RT-qPCR and Western blot analysis were conducted to detect the expression of NGF, BDNF and VEGF. Compared with the normal rats, the rats with DM displayed significantly decreased levels of NGF and BDNF, yet increased VEGF level (*p* < 0.05). The LV-miR-9 and LV-miR-29a groups exhibited lower levels of NGF and BDNF, while higher VEGF level than the LV-NC group (*p* < 0.05). As opposed to the LV-NC, LV-ISL1 + LV-miR-9 and LV-ISL1 + LV-miR-29a groups, the LV-ISL1 group showed upregulated expression of NGF and BDNF, but downregulated VEGF level. In comparison to the LV-ISL1 + LV-miR-9 group, the LV-miR-9 group exhibited decreased expression of NGF and BDNF, along with increased VEGF expression. In comparison with the LV-ISL1 + LV-miR-29a group, the LV-ISL1 group exhibited upregulated expression of NGF and BDNF, as well as downregulated VEGF expression (*p* < 0.05) (Figure 6B–6D). Further investigation was carried out to detect whether there were differences regarding expression patterns of NGF, BDNF, VEGF, miR-9 and miR-29a between peripheral blood of patients with DM and healthy participants. Results revealed upregulated miR-9, miR-29a and VEGF, along with downregulated NGF and BDNF in peripheral blood of patients with DM than that of healthy participants (*p* < 0.05) (Figure 6E–6G), which was exactly in line with the rat results.

It could be concluded that miR-9 and miR-29a aggravated the pathological changes in the expression of neurotrophic factors in the sciatic nerves, thus impairing the repair and regeneration functioning of the sciatic nerves by targeting ISL1.

## DISCUSSION

**Figure 7:**
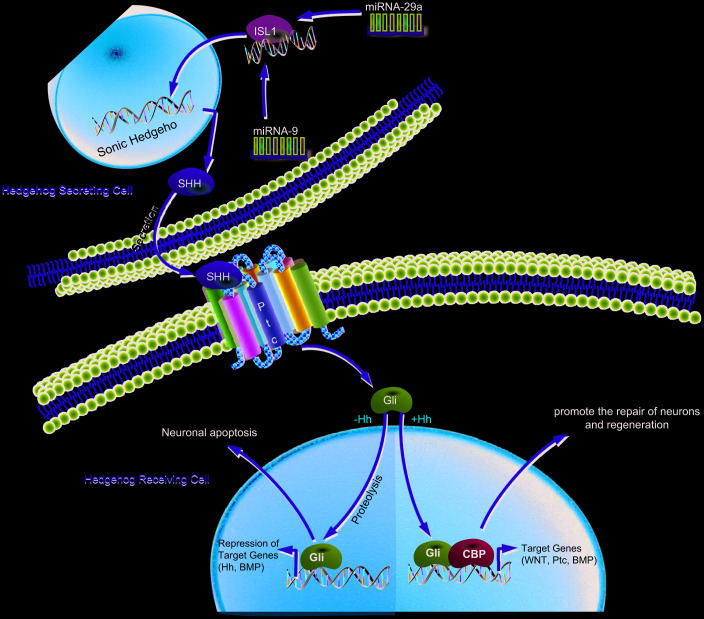
**Schematic illustration of the regulatory role of miR-9 and miR-29a in DPN.** miR-9 and miR-29a are highly expressed in DM, which inactivate the SHH signaling pathway by binding to ISL1, thereby promoting the occurrence and development of DPN.

Diabetic neuropathy is currently the most frequently occurring and medically challenging complication of DM and presents with the highest morbidity and mortality rates in addition to augmenting the economic burden of diabetic care [38]. DPN serves as one of the independent clinical predictors of cardiovascular mortality, with risk factors including hyperglycemia, smoking, dyslipidemia, and hypertension [39]. A recent study has shown that various miRs play essential roles in pancreatic islet development as well as insulin secretion, notably miR-9, which participates in the regulation of insulin secretion through interaction with Onecut-2 mRNA and has low expression in insulin-producing cells [40]. In the present study, we endeavored to explore the functional role of miR-9/miR-29a in DPN along with the underlying regulatory mechanism, finding that increased miR-9/miR-29a contributes to the occurrence and development of DPN via downregulation of ISL1 and inhibition of the SHH signaling pathway (Figure 7).

Our study found high expression of miR-9 and miR-29a and low expression of ISL1 in DM and further experimental data indicated that both miR-9 and miR-29a inhibited the expression of their target gene ISL1. miRs have been reported to play important roles in a variety of diseases including T2DM, and miR-9 and miR-29a have been known to be upregulated in serum of T2DM patients [41]. The miR-29 family, which is composed of miR-29a, b and c, greatly influences cell death, and is significantly expressed in patients with T1DM or T2DM as diabetic markers [42]. Similarly, miR-9 has been proposed as an independent risk biomarker of diabetic nephropathy, with elevated serum levels over the progression of the disease [43]. Moreover, overexpressed miR-29a results in defective glucose-induced insulin secretion, indicating the role of miR-29a in cytokine-regulated beta-cell dysfunction during early phases of T1DM [44]. Also, downregulation of miR-29a induced by Morin is beneficial in alleviating the diabetic condition by improving glucose metabolism and insulin signaling [45]. ISL1, a transcription factor, is associated with the development of the pancreas and in the differentiated functions of insulin-producing beta-cells [46]. ISL1 also combines with other LIM-homeodomain proteins to regulate the differentiation of various cells in the nervous system, indicating a general neurobiological role [47]. Another study shows that ISL1 plays a pivotal role in the development of motor neurons by regulating spinal and cranio-cervical motor neuron formation [48]. In addition, miR-9 plays a central role in specifying spinal motor neurons by targeting and suppressing Onecut1 (OC1), and thereby downregulates ISL1 expression [49].

Additionally, our study demonstrated that ISL1 mediated the SHH signaling pathway to improve DPN. ISL1 plays a fundamental role in the activation of insulin gene transcription of pancreatic beta-cells, and decreased ISL1 expression is related to the reduction of insulin expression regulated by dexamethasone [50]. Besides, SHH is essential for the formation of adult blood vessels and wound repair, and a deficiency of SHH in DM can lead to malfunctioning of angiogenic cells via the thrombospondin-1/CD36 signaling pathway [51]. The SHH signaling pathway is downregulated in cases of T1DM, and upregulation of this signaling pathway would not only mitigate the complications that present with DM, but can also rescue the functioning of endothelial progenitor cells and elevate neovascularization in T1DM [52]. Another study has indicated that SHH promotes angiogenesis as well as restoration of ischemic myocardium and skeletal muscles [53]. Moreover, SHH exerts neuroprotective effects on damaged retinal ganglion cells through Müller cells, as demonstrated in rat models of DM [54]. Also, the production of functional insulin-producing cells can be improved by regulating the SHH signaling pathway, which is regarded as a new approach for the cell-based treatment of T1DM [55]. In addition, we note that LIM homeodomain transcription factor ISL1 is required for upstream regulation of SHH [56]. According to a previous study, SHH can promote differentiation of motor neurons, which corresponds with the appearance of specific motor-neuron hallmarks, including ISL1 [57]. Intriguingly, insulin-like growth factor-1 (IGF-1) has been reported to be implicated in podocyte regeneration in diabetic nephropathy [58], suggesting its involvement in the pathology of DM. In addition, IGF signaling has been pointed out to mediate the SHH signaling pathway in regulating proliferation of cerebellar granule cell precursors [59]. Therefore, we inferred that other signaling cascades might be related to the ISL1-mediated SHH signaling pathway in the context of DM, which requires further investigation.

In conclusion, we found that miR-9 and miR-29a are highly expressed in DM, and inhibit the expression of ISL1 and the SHH signaling pathway, thereby promoting the occurrence and development of DPN, notably with respect to sciatic nerve pathology. Therefore, this study provides a key target for the treatment of DPN through the application of miR-9/miR-29a inhibitors. However, further studies are required to elucidate the specific mechanisms by which miR-9/miR-29a inhibitors alleviate DPN.

## MATERIALS AND METHODS

### Ethics statement

The study was approved by the Ethics Committee of Sichuan Academy of Medical Sciences and Sichuan Provincial People’s Hospital. The experiments involving human subjects were performed according to Declaration of Helsinki with written consents obtained from all participants prior to the study. All animal experiments were in line with the Guide for the Care and Use of Laboratory Animal by International Committees.

### Network meta-analysis

**Figure 8:**
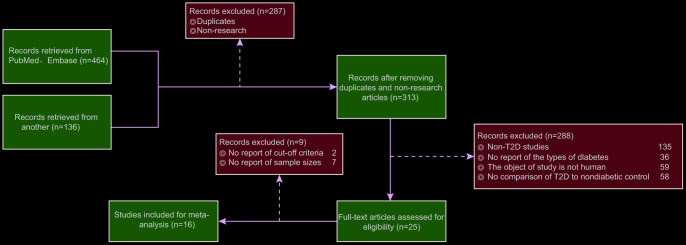
**Flow chart from identification of studies to final inclusion.** We initially retrieved 464 records from PubMed and Embase and 136 from other databases. Finally, 16 eligible studies are included for data collection and analysis.

We retrieved PubMed and Embase databases to obtain literature relevant to this study. The literature search was limited to the English language and ended in September 2017. The search terms included a combination of key words and terms as follows: diabetes, microRNA, microRNA-9, microRNA-29a, diabetic peripheral neuropathy, ISL1 and Sonic Hedgehog signaling pathway. Two researchers independently carried out data extraction based on a predefined form. Additionally, any disputes appearing during data extraction were resolved through discussion with multiple researchers. The inclusion criteria of the study were as follows: (1) published literature about miR expression and DM; (2) study design: case-control studies; (3) study subjects: patients diagnosed as T2DM; (4) literature with similar study design and methods; (5) complete literature data or literature data supporting calculations concordant with the results of the present report. The exclusion criteria were as follows: (1) literature without data integrity (such as non-case-control studies); (2) duplicate publications or documents with poor quality, insufficient information and unclear description of specific data; (3) conference reports, systematic reviews and summary articles; (4) studies unrelated to DM; (5) non-English language studies; (6) non-human studies. A total of 600 studies adhering to the search strategies were retrieved, as shown in Figure 8. Finally, a total of 16 publications were included in this study. The studies included blood samples of 972 diabetic patients and 860 healthy control patients (Table 1). The fold change method was used to calculate the fold change value of miR for each included reference, where Log_2_ Fold change = Log_2_ (miR expression in DM) - Log_2_ (miR expression in NC). Fold change value > 0 indicated that the miR was highly expressed in DM; fold change value < 0 indicated that the miR was poorly expressed in DM.

**Table 1:** A meta-analysis of miR expression profile in human DPN.

**Author**	**Year**	**Country**	**Number**		**Age (mean ± standard deviation, year)**		**Gender (male/female)**		**miR**
			**DM**	**NC**	**DM**	**NC**	**DM**	**NC**	
Zhao H ^26^	2010	China	40	15	69 ± 13	54 ± 14	17/23	6/9	miR-375
Jiang LQ ^27^	2013	Sweden	24	29	60.1 ± 5.0	60.9 ± 4.7	18/6	19/10	miR-98, let-7b, let-7c, let-7f, let-7g, and let-7i
Pescador N ^9^	2013	China	99	100	69.4 ± 7.12	42.9 ± 12.13	53/46	50/50	miR-138, miR-376a, miR-15b, and miR-503
Zhang T ^28^	2013	China	30	30	63 ± 8.56	61 ± 9	16/14	16/14	miR-126
Liu Y ^29^	2014	China	160	138	50.2 ± 6.7	46.7 ± 7.2	78/82	67/71	miR-126
Ortega FJ ^30^	2014	Spain	30	35	54 ± 10	48.1 ± 10.1	30/0	35/0	miR-140-5p, miR-142-3p, miR-222, miR-423-5p, miR-125b, miR-192, miR-195, miR-130b, miR-532-5p, and miR-126
Sun K ^31^	2014	China	100	100	51.33 ± 11.75	48.55 ± 12.41	54/46	44/56	miR-375
Yang Z ^32^	2014	China	24	20	51.13 ± 9.214	46.65 ± 16.181	16/8	8/12	miR-23a
Al-Kafaji G ^33^	2015	Arabian	24	24	52 ± 6	49 ± 9.1	10/14	13/11	miR-15a
Olivieri F ^34^	2015	Italy	117	76	66.51 ± 7.48	64.25 ± 7.56	69/48	36/40	MiR-21-5p and miR-126a-3p
Wu L ^35^	2015	China	25	20	46.7 ± 8.6	45.5 ± 10.2	-	-	miR-152, miR-17, miR-138, and miR-593
Dai X ^36^	2015	China	85	80	57.4 ± 9.6	59.5 ± 13.5	44/41	43/37	miR-483-5p, miR-19a, miR-29a, miR-20a, miR-24, and miR-25
Wang C ^37^	2016	China	92	92	47.7 ± 13.9	50.2 ± 14.2	58/34	56/36	miR-661, miR-571, miR-770-5p, miR-892b, and miR-1303
Wu K ^38^	2016	China	14	21	44 ± 6	42 ± 5	-	-	miR-126
Yan S ^39^	2016	China	50	50	46.22 ± 6.897	45.52 ± 6.215	27/23	22/28	miR-1249, miR-572, miR-1249, and miR-572
Yang S ^40^	2016	China	58	30	49.8 ± 9.1	48.2 ± 8.9	-	-	miR-144 and miR-223

Note: DPN, diabetic peripheral neuropathy; DM, diabetes mellitus; NC, normal control; miR, microRNA.

### Bioinformatics analysis

The Gene Expression Omnibus (http://www.ncbi.nlm.nih.gov/geo) was retrieved to download DM-related microarray datasets (GSE27382 and GSE95849) and annotation probe files. The datasets were obtained from the detection of Affymetrix GeneChip Mouse Genome 430 2.0 Array (CDF: Mouse4302_Mm_ENTREZG.cdf version 12.0.0) and Phalanx Human lncRNA One Array v1_mRNA. The Affy package of R software was employed for background correction and normalization of each dataset [60]. The linear model-the empirical Bayes statistical method in the Limma installation package, combined with the traditional *t*-test, was used for nonspecific filtration of expression data, to screen differentially expressed mRNAs [61]. The intersection genes of the differentially expressed genes from 2 datasets were found using a Venn map.

### Clinical sample collection

In total, serum samples from 30 patients diagnosed with DM in our hospital from June 2019 to October 2019 and from healthy volunteers (age: 42 – 75 years) were collected, respectively. Participants were excluded if (1) they suffered from common complications of DM, including retinopathy, nephropathy, and cardiovascular diseases; (2) they had been diagnosed as DM prior to our research; or (3) they had been on medication for 6 months.

### Cell culture and transfection

Human embryonic kidney cell line (HEK293T) (CoBioer Biosciences Co., Ltd., Nanjing, Jiangsu, China) was selected and sub-cultured with Dulbecco's Modified Eagle's Medium F12 culture solution containing 10% fetal bovine serum and penicillin/streptomycin (Procell Life Science and Technology Co., Ltd., Wuhan, Hubei, China) specially made for rat islet cells in an incubator at 37°C under 100% humidity with 5% CO_2_. The culture medium was renewed every 48 - 72 h. Prior to transfection, the cells in the logarithmic growth phase were seeded into a 6-well plate, with the cell density adjusted to a concentration of 2 × 10^5^ cells per well. Transfection was carried out using Lipofectamine^TM^-2000 (Invitrogen Inc., Carlsbad, CA, USA), strictly in accordance with the instructions of the kit. Plasmids were diluted in 50 μL serum-free Opti-Minimal Essential Medium (Opti-MEM) and gently mixed. Lipofectamine^TM^-2000 reagent of appropriate volume was diluted in Opti-MEM as well. After standing at room temperature for 5 min, the above two solutions were mixed and allowed to stand for another 20 min at room temperature. The mixture (100 μL) was then added and cultured for 48 h in an incubator at 37°C.

### Dual luciferase reporter gene assay

A biological prediction website was used to predict the targeting relationship between miR-9/miR-29a and ISL1. The dual luciferase reporter gene assay was carried out to determine whether ISL1 was the target of miR-9 and miR-29a. PCR was employed to amplify the sequence of ISL1 3’untranslated region (3’UTR). Double enzyme digestion was performed using Xho I and Not I restriction sites. The target fragment was cloned into the downstream of luciferase reporter gene pmirGLO (3577193, Promega, Madison, WI, USA). Site-directed mutagenesis was performed at the binding site of ISL1 and miR-9/miR-29a. miR-9 mimic, miR-29a mimic and corresponding NC were co-transfected into cells with the transfection reagent Lipofectamine^TM^-200 (Invitrogen), with at least 3 duplicate wells set in each group. Meanwhile, miR-9 inhibitor and miR-29a inhibitor were co-transfected with ISL1-wt and –mut reporter plasmids respectively, with at least 3 duplicate wells set in each group. Twenty-four hours after transfection, the cells were split and centrifuged at 12000 r/min for 1 min with the supernatant collected. The aforementioned operations were conducted in strict accordance with the instructions of the dual luciferase reporter assay system kit.

### Model establishment

A total of 100 healthy Sprague-Dawley male rats, with an average age of 5 weeks and weighing (180 ± 10) g, procured from Beijing Vital River Laboratory Animal Technology Co., Ltd., (Beijing, China) (animal license number: SCXK [Beijing] 2006-0009) were selected in our study. All rats were reared at the experimental animal center of Beijing University of Chinese Medicine under a specific pathogen free standard barrier system. The feeding conditions included temperature of (23 ± 2)°C, humidity of (55 ± 10)%, 12 h of illumination (7:00 - 19:00), and noise level < 60 dB. The rats were fed with free access to food and water in separate cages. After 1 week of adaptive feeding, 10 rats were randomly selected as the normal group (injected with normal saline) and fed with normal diet. The remaining 90 rats were fed with high-fat diet with 40% calories as fat (Beijing Hua Fukang Biological Technology Co., Ltd., Beijing, China). After 6 weeks, streptozotocin treatment was applied to establish the DM rat model. Streptozotocin (Sigma-Aldrich Chemical Company, St Louis, MO, USA) was mixed with 0.1 mmol/L sterile sodium citrate buffer (pH = 4.5, 4°C) to prepare a solution with a volume fraction of 0.45%, which was then injected into the left hypogastric cavity of rats (35 mg/kg). Then, 72 h after injection, tail-tip blood glucose of rats was measured using a blood glucose meter. Rats presenting with continuous blood glucose level > 16.7 mmol/L and stable blood glucose levels for 3 days were included for further experimentation. Except for 4 rats that failed modeling, the rest rats were successfully applied in the model establishment [62].

### Lentiviral infection

LV systems of ISL1, miR-9 and miR-29a were constructed. All primers used in the experiments were designed according to the sequences in Genebank and mirBase databases. The shuttle vector and expression vector were purchased from System Biosciences (CA, USA), and the primer sequences were synthesized by Shanghai Sangon Biological Engineering Technology and Services Co., Ltd. (Shanghai, China). The optimal multiplicity of infection in HEK293T cells infected with lentiviral vector was measured. After 72 h of infection with lentiviral vectors under optimal conditions, the virus was purified using CsCl density gradient centrifugation, and the titer of lentivirus was determined using the plaque formation method. The normal control was set as the normal group. A total of 80 DM model rats were assigned into the DM group (without infection), LV-NC group (infected with empty ISL1 LV), LV-ISL1 group (infected with overexpressed ISL1 LV), LV-ISL1 + SHH inhibit group (infected with overexpressed ISL1 LV and treated with the SHH signaling-inhibitor GDC-0449, 40 mg/Kg [63], Selleck Chemicals, Houston, TX, USA), LV-miR-9 group (infected with overexpressed miR-9 LV), LV-miR-29a group (infected with overexpressed miR-29a LV), LV-ISL1 + LV-miR-9 group (infected with overexpressed ISL1 and miR-9 LVs), and LV-ISL1 + LV-miR-29a group (infected with overexpressed ISL1 and miR-29a LVs), with 8 rats in each group. All rats were injected with 0.2 mL lentivirus diluent (concentration of 5 × 10^9^) via tail vein every day for 14 days on a high-fat diet.

### Serum extraction and protein quantification

Blood was collected from rats in each group by cutting their tails and placed in an evacuated heparinized tube on ice for 30 min. The serum in the supernatant was collected through centrifugation at 4000 r/min for 10 min and stored in a refrigerator at -80°C for further quantification of SHH, GLI1 and PTCH.

### OGTT

OGTT was conducted at the 12^th^ week following the successful establishment a DM rat model. After 12 h of overnight fasting, the end of the rat tail (1 - 2 mm) was excised and gently pressed to force its blood into one drop. A standard glucometer was used to detect FBG, which was recorded as the blood glucose level at 0 min. Next, rats were fed with 20% glucose (2 g/kg), and blood glucose level was measured by glucometer at 15, 30, 45, 60, 90 and 120 min, respectively.

### HOMA-IR

Rat eyeball was extracted at the end of the 4^th^ and 12^th^ week to collect blood and determine FBG. According to the instructions of an insulin enzyme linked immunosorbent assay kit, plasma insulin level was determined, with two duplicate wells set in each sample. HOMA-IR formula was as follows: FBG (mmol/L) × insulin (μU/mL)/22.5.

### AR activity detection

Rat sciatic nerves were collected, washed by cold normal saline, weighed and added with 1.5 mL cold normal saline for homogenate. The homogenate was centrifuged at 3500 r/min at 0 - 4°C for 15 min. The supernatant was then collected for AR activity detection using fluorescence spectrophotometry.

### RT-qPCR

Sciatic nerves of rats cryopreserved at -80°C from each group were selected to extract total RNA using a Trizol reagent (Gibco Company, Grand Island, NY, USA). PCR primers were designed according to the sequences in Genbank (Table 2) and synthesized by Shanghai Sangon Biological Engineering Technology and Services Co., Ltd. (Shanghai, China). The concentration and purity of total RNA were determined using an ultraviolet spectrophotometer. U6 was regarded as the internal reference of miR-9 and miR-29a, and glyceraldehyde-3-phosphate dehydrogenase (GAPDH) was used as the internal reference of ISL1, SHH, GLI1, PTCH, NGF, BDNF, and VEGF. The 2^-ΔΔCT^ method was used to calculate the relative expression of the target genes.

**Table 2:** Primer sequences for RT-qPCR.

**Gene**	**Sequence (5’– 3’)**
miR-9	F: ATAAAGCTAGATAACCGAAAGT
	R: TTGGGGTGCTCGTGCAGATCGAA
miR-29a	F: ACACTCCAGCTGGGTAGCACCATCTGAAATC
	R: TGGTGTCGTGGAGTCG
ISL1	F: TGCCCGCTCCAAGGTGTA
	R: CCGA AGCGCAAATTCGTC
SHH	F: CTGGCCAGATGTTTT CTGGT
	R: TAAAGGGGTCAGCTTTTTGG
GLI1	F: AGTGCTTGCC CAAAGGATGA
	R: TGACGTGAGACCTGA TCTCGTT
PTCH	F: ATGCTGGAGGAGAACAAGCAA
	R: CGAGGA CCCCATCATCAGAT
NCF	F: ACC TCT TCG GAC ACT CTG GA
	R: GTC CGT GGC TGT GGT CTT AT
BDNF	F: GGT CAC AGT CCT GGA GAA AG
	R: GTC TAT CCT TAT GAA CCG CC
VEGF	F: GGA CAT CTT CCA GGA GTA CC
	R: CGC ATG ATC TGC ATA GTG AC
U6	F: ATGACGTCTGCCTTGGAGAAC
	R: TCAGTGTGCTACGGAGTTCAG
GADPH	F: CTGACATGCCGCCTGGAGA
	R: ATGTAGGCCATGAGGTCCAC

Note: RT-qPCR, reverse transcription quantitative polymerase chain reaction; miR, microRNA; ISL1, Islet factor 1; SHH, Sonic Hedgehog; GLI1, glioma-associated oncogene homolog 1; PTCH, patched tumor suppressor gene; GADPH, glyceraldehyde-phosphate dehydrogenase; NGF, nerve growth factor; BDNF, brain-derived neurotrophic factor; VEGF, vascular endothelial growth factor; F, forward; R, reverse.

### Western blot analysis

Sciatic nerves cryopreserved at -80°C from each group were selected and weighed using an electronic balance. The tissues were cut using ophthalmological incision scissors in the mortar. Appropriate amounts of phenyl sulfonyl fluoride and liquid nitrogen were added to the tissues, followed by uniform grinding using a homogenizer and the addition of protein extract (500 μL/100 mg). Then, the tissues were incubated and centrifuged to obtain the supernatant. A bicinchoninic acid protein quantitative kit (Catalog No.23225, Pierce, Rockford, IL, USA) was used to determine the protein concentration of the tissues. The protein was boiled for 5 min, separated using polyacrylamide gel electrophoresis at proper concentrations, and transferred onto a polyvinylidene fluoride membrane. Subsequently, the membrane was sealed using 5% skim milk at room temperature for 1 h and rinsed with Tris-buffered saline Tween 20 (TBST). Then, the membrane was incubated at 4°C overnight with the following primary antibodies: rabbit anti-mouse polyclonal antibodies to SHH (1:500, ab19897), GLI1 (1:3000, ab151796), PTCH (1:500, PA1-46222), NGF (1:2000, ab6199), BDNF (1:500, ab10505), and VEGF (1:2000, ab1316). All antibodies except PTCH were purchased from the Abcam (Cambridge, MA, USA), and PTCH was purchased from Thermo Fisher Scientific (CA, USA). The membrane was rinsed with TBST, and then incubated at room temperature for 2 h with horseradish peroxidase-labeled goat anti-rabbit immunoglobulin G antibody diluted using 5% skimmed milk (1:1000, ab97091, Abcam), followed by TBST washing again. A 3,3’-diaminobenzidine (DAB) kit (Beijing Solarbio Science and Technology Co., Ltd., Beijing, China) was used for coloration. The software Quantity one was employed to analyze the band gray value. The difference of protein expression between two groups was compared and recorded.

### Determination of serum biochemical indexes

The rats were anesthetized with 3% pentobarbital and blood was collected from the carotid artery, followed by serum separation. Various biochemical indexes including FBG, TC, TG and FFA were measured and recorded. In addition, the left epididymal fat was weighed. FBG of the rats was measured using a fast blood glucose meter. TC and TG were determined using the enzyme method on the automatic biochemical analyzer. Serum insulin was determined by radioimmunoassay and blood FFA was determined by the copper coloration method (Nanjing Jiancheng Bioengineering Institute, Nanjing, China).

### Motor nerve conduction velocity

Motor nerve conduction velocity in rats was measured by means of the electrode insertion method. The rats were anesthetized and then placed in the prone position. The stimulating electrode, recording electrode, and reference electrode were fixed at the right sciatic notch of rats, the second toe of the right foot, and the midpoint between the stimulating electrode and the recording electrode, respectively. The stimulus wave was a mono-pulse square wave with a pulse width of 0.1 ms, and the stimulus intensity was 1.5 times the threshold and intervals between two stimuli was more than 5 s. The time of the potential action from the stimulus nerve to the distal muscle was recorded, namely, the latency period. The measurement was repeated 5 times and the average value was recorded. Then, the distance between the stimulating electrode and the recording electrode was measured. Motor nerve conduction velocity (m/s) = distance between stimulating electrode and recording electrode/latency period.

### Withdrawal threshold of thermal pain and mechanical pain

Thermal nociception threshold was detected using a YLS-6B hot plate apparatus. The hot plate was heated to a temperature of 55°C and the sole of the rat’s feet was placed on the hot plate. The rats were stimulated by heating and then the obvious foot licking actions were regarded as a response index of nociception. The time required to induce the nociception response was regarded as the WTL. Mechanical nociception threshold was measured using a YLS-3E tenderness tester. The rats were fixed and their tails were pressed/pressurized 3 cm from the tip of the tail. In addition, rat vocalization induced by nociception was used as a response index of nociception. The pressure value (g) was regarded as the WMT. After modeling, the rats were tested for WTL and WMT once a week during the time of lentiviral infection.

### HE staining

Sciatic nerves fixed using 4% paraformaldehyde were placed in gradient ethanol, dehydrated and cleared in xylene. The completely cleared nerve tissues were immersed in soft wax and then hard wax for 1.5 h, respectively, followed by embedding in paraffin and slicing into 5-μm slices. Then, the slices were flattened in a water bath heated to 37°C. Subsequently, the flattened slices were attached to the slides and dried for 4 h. The dried sciatic nerve slices were immersed in xylene for clearing, and then hydrated in a gradient ethanol solution. The completely hydrated slices were cleared off the surrounding moisture, followed by HE staining (Beijing Zhongshan Jinqiao Biotechnology Co., Ltd., Beijing, China). After dehydration by gradient ethanol and clearing with xylene again, the slices were sealed using neutral balsam, observed and photographed under a light microscope.

### Immunohistochemistry

The sciatic nerves were fixed, sectioned with paraffin, and dewaxed by xylene and gradient ethanol to water. Then, the sections were incubated in 0.3% hydrogen peroxide methanol solution for 30 min, followed by treatment with 0.3% Triton X-100 for 30 min. Next, the sections were subjected to antigen repair under microwave for 2 h, sealed using a normal goat serum solution, and incubated with primary antibody, namely the rabbit anti-rat NGF, BDNF, and VEGF antibodies (1:200) at 4°C overnight. The subsequent day, the sections were added with secondary antibody goat anti-rabbit antibody (1:200), followed by rinsing with 0.01 mol/L PBS 3 times, 5 min each. The sections were then colored using DAB, dehydrated by gradient ethanol, immersed into xylene, and sealed using neutral balsam. Finally, the sections were photographed under a light microscope to count the number of positive cells. The ratio of positive cells to total cells was counted and recorded in each section.

### Ultrastructural observation under an electron microscope

The rats were fixed in supine positions after anesthetized by 10% chloral hydrate (300 mg/kg). After clearing the surrounding tissues and exposing the heart, the right atrial appendage was cut open and normal saline heated to 37°C was injected into the left ventricle until the effluent liquid was colorless. The sciatic nerves were then exposed and cut from the sciatic notch and the crotch of tibial and peroneal nerves, at both ends of each about 1 - 2 mm, splitting it from the middle. One part of the extracted nerves was fixed in glutaraldehyde, and another part was fixed with 4% paraformaldehyde. After fixed with 4% paraformaldehyde, the sciatic nerves were embedded with paraffin and sectioned. The 4% glutaraldehyde fixed sections were further incubated at 4°C overnight. Then, the sections were fixed in 1% osmic acid and dehydrated using gradient ethanol with concentrations as follows: 25%, 35%, 50%, 70%, 75%, 80%, and 95%, each for 5 min, and 100% ethanol for 10 min. Following that, the dehydrated sections were embedded in Epon embedding agent and sliced into ultrathin sections. Ultrastructural changes of sciatic nerves in each group were observed using a transmission electron microscope (LVEM5, Delong Instruments, Czech Republic).

### Statistical analysis

Statistical analyses were performed using the SPSS 19.0 statistical software (IBM Corp., Armonk, NY, USA). Measurement data were presented as mean ± standard deviation. Data between two groups with normal distribution were examined by *t*-test, and non-normal distribution by Mann-Whitney U. Data among multiple groups were analyzed using one-way analysis of variance and Student-Newman-Keuls method was utilized for pairwise comparison. A value of *p* < 0.05 was considered to be statistically significant.
